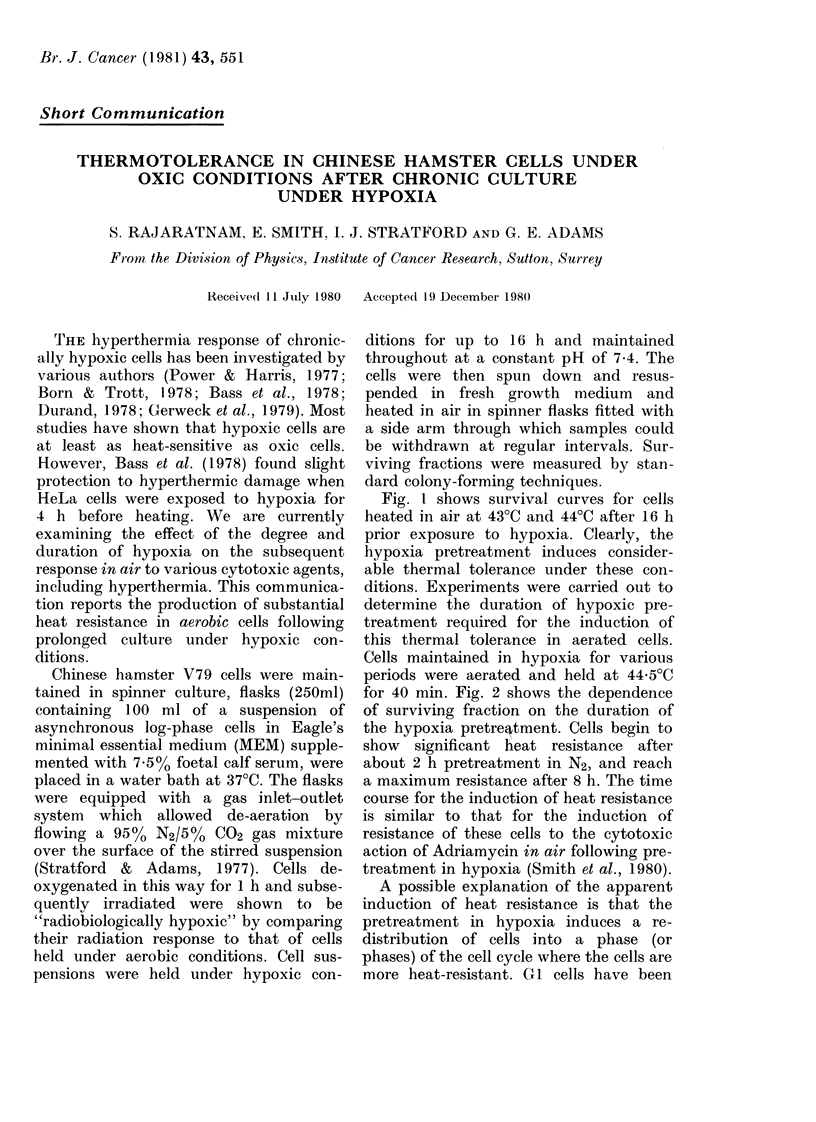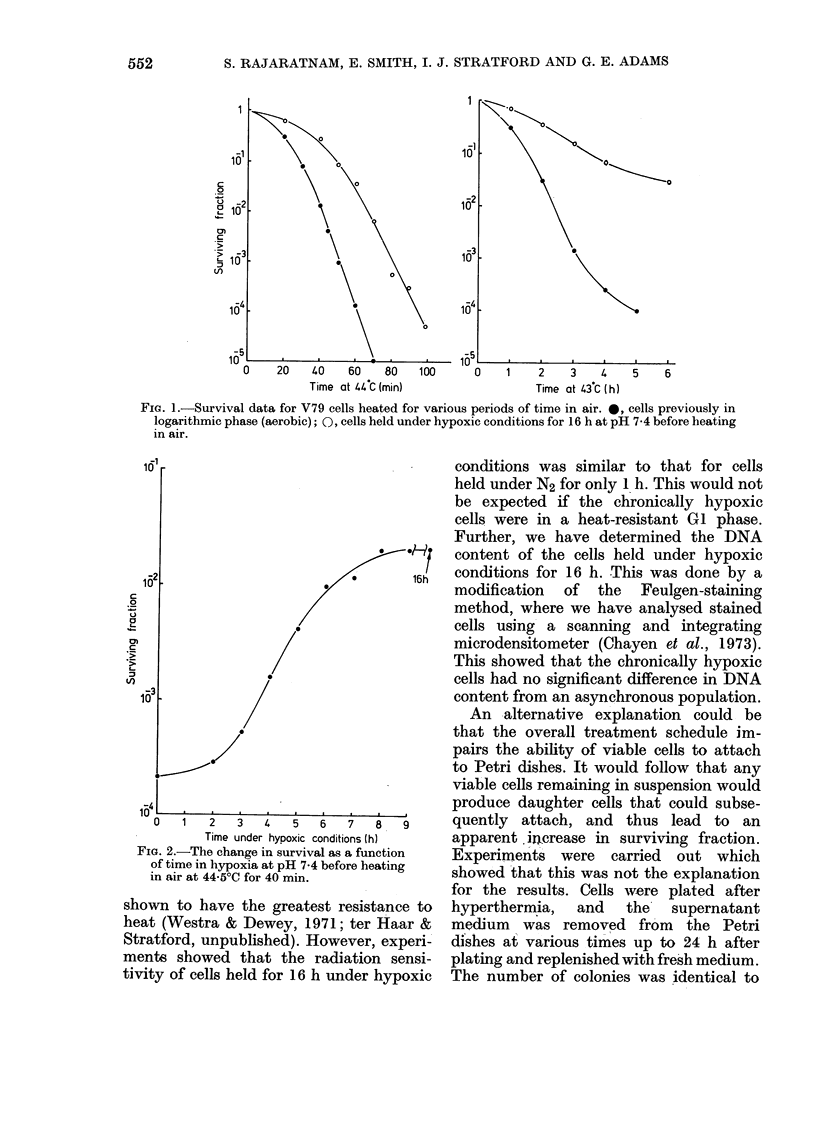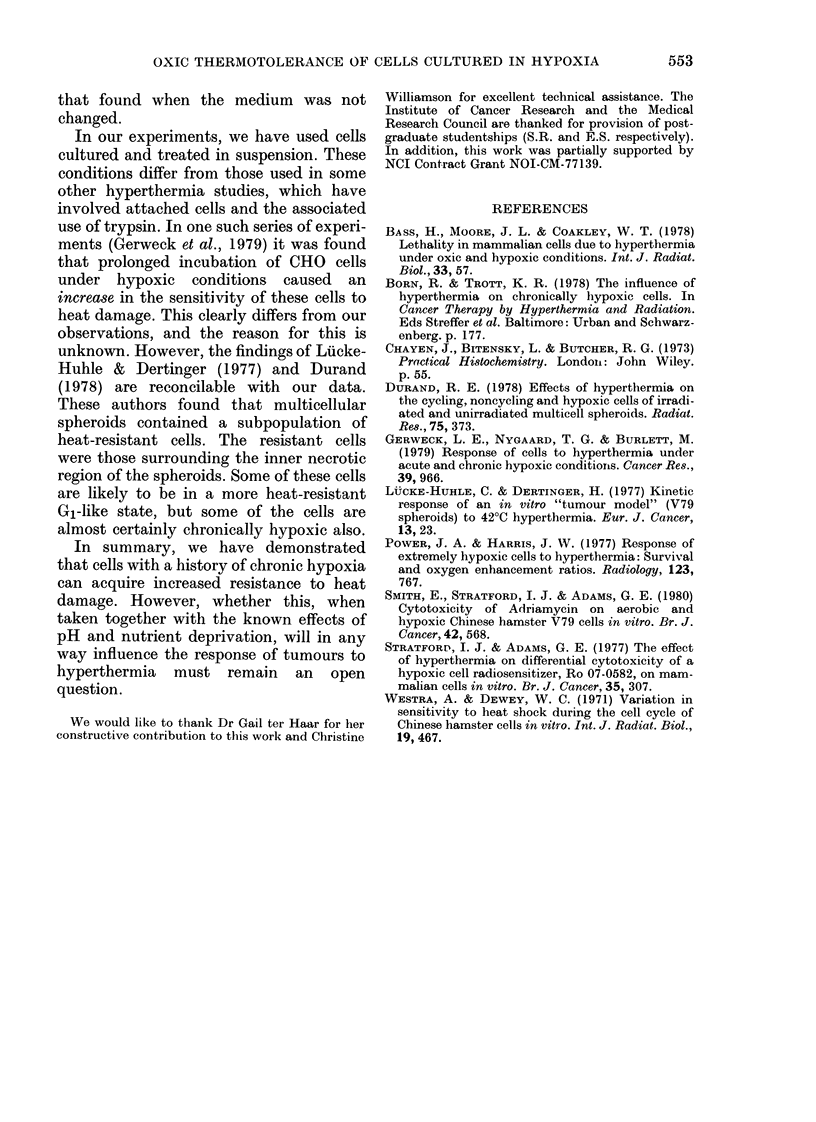# Thermotolerance in chinese hamster cells under oxic conditions after chronic culture under hypoxia.

**DOI:** 10.1038/bjc.1981.80

**Published:** 1981-04

**Authors:** S. Rajaratnam, E. Smith, I. J. Stratford, G. E. Adams


*Br. J. Cancer* (1981) **43**, 551

***Short Communication***

### THERMOTOLERANCE IN CHINESE HAMSTER CELLS UNDER OXIC CONDITIONS AFTER CHRONIC CULTURE UNDER HYPOXIA

S. RAJARATNAM, E. SMITH, I. J. STRATFORD AND G. E. ADAMS

*From the Division of Physics, Institute of Cancer Research, Sutton, Surrey*

Received 11 July 1980 Accepted 19 December 1980

THE hyperthermia response of chronically hypoxic cells has been investigated by various authors (Power & Harris, 1977; Born & Trott, 1978; Bass *et al.*, 1978; Durand, 1978; Gerweck *et al.*, 1979). Most studies have shown that hypoxic cells are at least as heat-sensitive as oxic cells. However, Bass *et al.* (1978) found slight protection to hyperthermic damage when HeLa cells were exposed to hypoxia for 4 h before heating. We are currently examining the effect of the degree and duration of hypoxia on the subsequent response *in air* to various cytotoxic agents, including hyperthermia. This communication reports the production of substantial heat resistance in *aerobic* cells following prolonged culture under hypoxic conditions.

Chinese hamster V79 cells were maintained in spinner culture, flasks (250ml) containing 100 ml of a suspension of asynchronous log-phase cells in Eagle's minimal essential medium (MEM) supplemented with 7·5% foetal calf serum, were placed in a water bath at 37°C. The flasks were equipped with a gas inlet–outlet system which allowed de-aeration by flowing a 95% $N_2$/5% $CO_2$ gas mixture over the surface of the stirred suspension (Stratford & Adams, 1977). Cells deoxygenated in this way for 1 h and subsequently irradiated were shown to be "radiobiologically hypoxic" by comparing their radiation response to that of cells held under aerobic conditions. Cell suspensions were held under hypoxic conditions for up to 16 h and maintained throughout at a constant pH of 7·4. The cells were then spun down and resuspended in fresh growth medium and heated in air in spinner flasks fitted with a side arm through which samples could be withdrawn at regular intervals. Surviving fractions were measured by standard colony-forming techniques.

Fig. 1 shows survival curves for cells heated in air at 43°C and 44°C after 16 h prior exposure to hypoxia. Clearly, the hypoxia pretreatment induces considerable thermal tolerance under these conditions. Experiments were carried out to determine the duration of hypoxic pretreatment required for the induction of this thermal tolerance in aerated cells. Cells maintained in hypoxia for various periods were aerated and held at 44·5°C for 40 min. Fig. 2 shows the dependence of surviving fraction on the duration of the hypoxia pretreatment. Cells begin to show significant heat resistance after about 2 h pretreatment in $N_2$, and reach a maximum resistance after 8 h. The time course for the induction of heat resistance is similar to that for the induction of resistance of these cells to the cytotoxic action of Adriamycin *in air* following pretreatment in hypoxia (Smith *et al.*, 1980).

A possible explanation of the apparent induction of heat resistance is that the pretreatment in hypoxia induces a redistribution of cells into a phase (or phases) of the cell cycle where the cells are more heat-resistant. G1 cells have been

FIG. 1.—Survival data for V79 cells heated for various periods of time in air. ●, cells previously in logarithmic phase (aerobic); ○, cells held under hypoxic conditions for 16 h at pH 7·4 before heating in air.

FIG. 2.—The change in survival as a function of time in hypoxia at pH 7·4 before heating in air at 44·5°C for 40 min.

shown to have the greatest resistance to heat (Westra & Dewey, 1971; ter Haar & Stratford, unpublished). However, experiments showed that the radiation sensitivity of cells held for 16 h under hypoxic conditions was similar to that for cells held under $N_2$ for only 1 h. This would not be expected if the chronically hypoxic cells were in a heat-resistant G1 phase. Further, we have determined the DNA content of the cells held under hypoxic conditions for 16 h. This was done by a modification of the Feulgen-staining method, where we have analysed stained cells using a scanning and integrating microdensitometer (Chayen *et al.*, 1973). This showed that the chronically hypoxic cells had no significant difference in DNA content from an asynchronous population.

An alternative explanation could be that the overall treatment schedule impairs the ability of viable cells to attach to Petri dishes. It would follow that any viable cells remaining in suspension would produce daughter cells that could subsequently attach, and thus lead to an apparent increase in surviving fraction. Experiments were carried out which showed that this was not the explanation for the results. Cells were plated after hyperthermia, and the supernatant medium was removed from the Petri dishes at various times up to 24 h after plating and replenished with fresh medium. The number of colonies was identical to that found when the medium was not changed.

In our experiments, we have used cells cultured and treated in suspension. These conditions differ from those used in some other hyperthermia studies, which have involved attached cells and the associated use of trypsin. In one such series of experiments (Gerweck *et al.*, 1979) it was found that prolonged incubation of CHO cells under hypoxic conditions caused an *increase* in the sensitivity of these cells to heat damage. This clearly differs from our observations, and the reason for this is unknown. However, the findings of Lücke-Huhle & Dertinger (1977) and Durand (1978) are reconcilable with our data. These authors found that multicellular spheroids contained a subpopulation of heat-resistant cells. The resistant cells were those surrounding the inner necrotic region of the spheroids. Some of these cells are likely to be in a more heat-resistant $G_1$-like state, but some of the cells are almost certainly chronically hypoxic also.

In summary, we have demonstrated that cells with a history of chronic hypoxia can acquire increased resistance to heat damage. However, whether this, when taken together with the known effects of pH and nutrient deprivation, will in any way influence the response of tumours to hyperthermia must remain an open question.

We would like to thank Dr Gail ter Haar for her constructive contribution to this work and Christine Williamson for excellent technical assistance. The Institute of Cancer Research and the Medical Research Council are thanked for provision of post-graduate studentships (S.R. and E.S. respectively). In addition, this work was partially supported by NCI Contract Grant NOI-CM-77139.

### REFERENCES

BASS, H., MOORE, J. L. & COAKLEY, W. T. (1978) Lethality in mammalian cells due to hyperthermia under oxic and hypoxic conditions. *Int. J. Radiat. Biol.*, **33**, 57.

BORN, R. & TROTT, K. R. (1978) The influence of hyperthermia on chronically hypoxic cells. In *Cancer Therapy by Hyperthermia and Radiation.* Eds Streffer *et al.* Baltimore: Urban and Schwarzenberg. p. 177.

CHAYEN, J., BITENSKY, L. & BUTCHER, R. G. (1973) *Practical Histochemistry.* London: John Wiley. p. 55.

DURAND, R. E. (1978) Effects of hyperthermia on the cycling, noncycling and hypoxic cells of irradiated and unirradiated multicell spheroids. *Radiat. Res.*, **75**, 373.

GERWECK, L. E., NYGAARD, T. G. & BURLETT, M. (1979) Response of cells to hyperthermia under acute and chronic hypoxic conditions. *Cancer Res.*, **39**, 966.

LÜCKE-HUHLE, C. & DERTINGER, H. (1977) Kinetic response of an *in vitro* "tumour model" (V79 spheroids) to 42°C hyperthermia. *Eur. J. Cancer*, **13**, 23.

POWER, J. A. & HARRIS, J. W. (1977) Response of extremely hypoxic cells to hyperthermia: Survival and oxygen enhancement ratios. *Radiology*, **123**, 767.

SMITH, E., STRATFORD, I. J. & ADAMS, G. E. (1980) Cytotoxicity of Adriamycin on aerobic and hypoxic Chinese hamster V79 cells *in vitro. Br. J. Cancer*, **42**, 568.

STRATFORD, I. J. & ADAMS, G. E. (1977) The effect of hyperthermia on differential cytotoxicity of a hypoxic cell radiosensitizer, Ro 07-0582, on mammalian cells *in vitro. Br. J. Cancer*, **35**, 307.

WESTRA, A. & DEWEY, W. C. (1971) Variation in sensitivity to heat shock during the cell cycle of Chinese hamster cells *in vitro. Int. J. Radiat. Biol.*, **19**, 467.